# Polyamine Anabolism Promotes Chemotherapy‐Induced Breast Cancer Stem Cell Enrichment

**DOI:** 10.1002/advs.202404853

**Published:** 2024-07-26

**Authors:** Guangyu Ji, Jia Liu, Zhiqun Zhao, Jie Lan, You Yang, Zheng Wang, Huijing Feng, Kai Ji, Xiaofeng Jiang, Huize Xia, Guangyao Wei, Yajing Zhang, Yuhong Zhang, Xinlong Du, Yawen Wang, Yuanyuan Yang, Zhaojian Liu, Kai Zhang, Qi Mei, Rong Sun, Haiquan Lu

**Affiliations:** ^1^ The Second Hospital and Advanced Medical Research Institute Cheeloo College of Medicine Shandong University Jinan 250012 China; ^2^ School of Basic Medical Sciences Cheeloo College of Medicine Shandong University Jinan 250012 China; ^3^ Department of Radiation Oncology Cancer Center and State Key Laboratory of Biotherapy West China Hospital Sichuan University Chengdu 610041 China; ^4^ Department of Pediatrics (Children Hematological Oncology) Birth Defects and Childhood Hematological Oncology Laboratory The Affiliated Hospital of Southwest Medical University Sichuan Clinical Research Center for Birth Defects Luzhou 646000 China; ^5^ Department of Urology Shandong Provincial Hospital Affiliated to Shandong First Medical University Jinan 250021 China; ^6^ Cancer Center, Shanxi Bethune Hospital Shanxi Academy of Medical Sciences Tongji Shanxi Hospital Third Hospital of Shanxi Medical University Taiyuan 030032 China; ^7^ Shandong Helix Matrix Data Technology Jinan 250014 China; ^8^ Youth League Committee Qilu Hospital Shandong University Jinan 250012 China; ^9^ Department of Breast Surgery, General Surgery Qilu Hospital Shandong University Jinan 250012 China; ^10^ Shandong Artificial Intelligence Institute Qilu University of Technology (Shandong Academy of Sciences) Jinan 250399 China; ^11^ Department of Oncology, Tongji Hospital Tongji Medical College Huazhong University of Science and Technology Wuhan 430000 China; ^12^ Key Laboratory for Experimental Teratology of the Ministry of Education Cheeloo College of Medicine Shandong University Jinan 250012 China; ^13^ Center for Reproductive Medicine Shandong University Jinan 250001 China

**Keywords:** breast cancer stem cell, britannin, chemotherapy, HIF‐1 inhibitor, hypoxia‐inducible factor 1, polyamine anabolism

## Abstract

Breast cancer patients may initially benefit from cytotoxic chemotherapy but experience treatment resistance and relapse. Chemoresistant breast cancer stem cells (BCSCs) play a pivotal role in cancer recurrence and metastasis, however, identification and eradication of BCSC population in patients are challenging. Here, an mRNA‐based BCSC signature is developed using machine learning strategy to evaluate cancer stemness in primary breast cancer patient samples. Using the BCSC signature, a critical role of polyamine anabolism in the regulation of chemotherapy‐induced BCSC enrichment, is elucidated. Mechanistically, two key polyamine anabolic enzymes, ODC1 and SRM, are directly activated by transcription factor HIF‐1 in response to chemotherapy. Genetic inhibition of HIF‐1‐controlled polyamine anabolism blocks chemotherapy‐induced BCSC enrichment in vitro and in xenograft mice. A novel specific HIF‐1 inhibitor britannin is identified through a natural compound library screening, and demonstrate that coadministration of britannin efficiently inhibits chemotherapy‐induced HIF‐1 transcriptional activity, ODC1 and SRM expression, polyamine levels, and BCSC enrichment in vitro and in xenograft and autochthonous mouse models. The findings demonstrate the key role of polyamine anabolism in BCSC regulation and provide a new strategy for breast cancer treatment.

## Graphical abstract

A critical role of polyamine anabolism in the regulation of chemotherapy‐induced breast cancer stem cell enrichment, is elucidated. Chemotherapy induces the expression of two key polyamine anabolic enzymes, ODC1 and SRM, in a HIF‐1‐dependent manner. Co‐administration of britannin, a novel specific HIF‐1 inhibitor, efficiently inhibits chemotherapy‐induced polyamine anabolism and breast cancer stem cell enrichment.

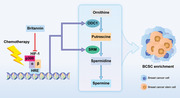

## 1 Introduction

Cytotoxic chemotherapy is a widely‐used systemic treatment for early‐stage and advanced breast cancer patients.^[^ ^1^, ^2^ ^]^ Although many patients initially respond to chemotherapy, they often experience tumor recurrence and metastasis.^[^ ^3^ ^]^ The existence of breast cancer stem cells (BCSCs), a small subpopulation of breast cancer cells that possess infinite proliferative potential and tumor‐initiating capacity, is the major reason for chemoresistance and tumor relapse.^[^ ^4^, ^5^, ^6^, ^7^, ^8^ ^]^ Recent studies have illustrated the plasticity of breast cancer cells that chemotherapy induces transformation from non‐BCSCs to BCSCs, which further increases the risk of tumor recurrence and metastasis.^[^ ^9^, ^10^, ^11^ ^]^ Therefore, a better understanding of BCSC regulation in response to chemotherapy are urgently needed for the improvement of breast cancer patient outcome.

One major challenge in targeting BCSCs is the identification of BCSCs in vivo. Xenotransplantation assays are considered as the gold standard for measuring BCSCs by definition.^[^ ^6^, ^12^ ^]^ The development of biomarkers, such as expression of ESA/CD44 and absence of expression of CD24,^[^ ^13^ ^]^ and activity of aldehyde dehydrogenase (ALDH),^[^ ^14^ ^]^ facilitates in vitro enrichment and characterization of BCSCs. However, non‐identical population of breast cancer cells are identified by using different markers,^[^ ^15^, ^16^ ^]^ suggesting that none of these single markers defines the BCSC population accurately. Lack of precise biomarkers further complicates identification of the BCSC population in vivo, which is a major obstacle in targeting BCSCs specifically. In addition, BCSCs enriched from bulk breast cancer cells experience fast differentiation and exhaustion during cell culture, making it difficult to study the characteristics of BCSCs in vitro. Therefore, discovering a clinically feasible approach to identify BCSCs will help to improve treatment outcome in the perspective of eradicating BCSCs.

BCSCs display a distinctive metabolic phenotype that differs from bulk cancer cells but shares similarity with normal stem cells.^[^ ^17^, ^18^ ^]^ A growing body of literature indicates that metabolic changes contribute to the regulation of the BCSC phenotype rather than being a mere consequence.^[^ ^19^, ^20^, ^21^ ^]^ The role of glucose metabolic pathways in the regulation of the BCSC phenotype has been extensively studied.^[^ ^22^, ^23^ ^]^ Recently, polyamine metabolism is implied in the regulation of BCSCs because of its interplay with other BCSC‐regulating pathways, such as MYC, PI3K‐AKT‐mTOR, AMPK, and RAS signaling pathways.^[^ ^24^, ^25^, ^26^, ^27^ ^]^ Polyamines, which includes putrescine, spermidine, and spermine, are polycationic alkylamines that are involved in many fundamental biological processes of cell growth and survival.^[^ ^28^, ^29^ ^]^ Increased polyamine requirement and dysregulated polyamine metabolism have been observed in breast cancer, making it a potential therapeutic target.^[^ ^30^, ^31^ ^]^ However, the (dys)regulation of polyamine metabolism in breast cancer, the precise role of polyamine metabolism in regulating BCSCs, and its underlying mechanisms are largely elusive.

In the present study, we developed an mRNA expression‐based BCSC signature from primary breast cancer patient samples to easily approach cancer stemness in vivo. Using this BCSC signature, we found that chemotherapy induces BCSC enrichment, which is dependent on hypoxia‐inducible factor 1 (HIF‐1)‐controlled polyamine anabolism. We also developed a specific HIF‐1 inhibitor, britannin, that effectively inhibits polyamine anabolism and eradicates BCSCs.

## 2 Results

### 2.1 Development and Validation of an mRNA‐Based BCSC Signature

**Figure 1:**
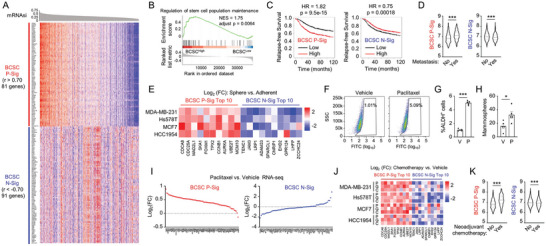
An mRNA expression‐based BCSC signature demonstrates chemotherapy‐induced BCSC enrichment. A) Development of BCSC signature. B,C) GSEA and Kaplan–Meier analysis of relapse‐free survival was performed based on BCSC P‐Sig and N‐Sig expression in patient samples from TCGA BRCA. D) BCSC P‐Sig and N‐Sig expression in primary tumor tissue from metastatic (Yes) versus non‐metastatic (No) cancer within 5 years in METABRIC was compared. E) Adherent or sphere breast cancer cells were cultured and qPCR assay was performed for the mRNA expression of top 10 BCSC P‐Sig and N‐Sig genes. F‐I) SCID mice were injected with MDA‐MB‐231 cells and treated with vehicle (V) or paclitaxel (P). ALDH (F, G), mammosphere (H), and RNA sequencing (I) assays were performed. J) Breast cancer cell lines were treated with vehicle, paclitaxel (P), gemcitabine (G), or carboplatin (C) for 3 days and qPCR assay was performed. K) BCSC P‐Sig and N‐Sig expression in primary tumor tissue from patients who received neoadjuvant chemotherapy (Yes) and who did not receive chemotherapy (No) in METABRIC was compared. ^*^ *p* < 0.05, ^***^ *p* < 0.001.

To quantitatively evaluate stemness of primary breast cancer patient samples at gene expression level, we developed an mRNA‐based BCSC signature using data from the Cancer Genome Atlas (TCGA) breast invasive carcinoma (BRCA) dataset, by performing Pearson correlation analysis between mRNA expression and a one‐class logistic regression (OCLR) machine‐learning algorithm‐based mRNA stemness index (mRNAsi).^[^ ^32^ ^]^ We identified 81 genes that have Pearson r > 0.70 and 91 genes that have Pearson r < −0.70, and defined them as BCSC positive signature (P‐Sig) and negative signature (N‐Sig), respectively (**Figure** **1**A; Table S7, Supporting Information). We performed rank‐based gene set enrichment analysis (GSEA) by comparing BCSC^high^ and BCSC^low^ groups, which was stratified by the expression of the BCSC signature, and found that BCSC signature genes were enriched in “Regulation of stem cell population maintenance” (Figure 1B). The mRNA‐based BCSC signature we developed also had strong correlation with a previously established BCSC signature in the literature^[^ ^33^ ^]^ (Figure S1A, Supporting Information). To determine the clinical performance of the BCSC signature, we analyzed the correlation between BCSC signature expression and relapse‐free survival (RFS) of 2032 patients. Patients with BCSC P‐Sig expression above the median had decreased RFS, whereas patients with BCSC N‐Sig expression above the median had increased RFS (Figure 1C). In addition, BCSC P‐Sig expression was higher, while BCSC N‐Sig expression was lower, in breast cancer tissues compared with normal breast tissues (Figure S1B and Table S8, Supporting Information). Among different subtypes of breast cancer, basal‐like breast cancers exhibited the highest BCSC P‐Sig and the lowest BCSC N‐Sig expression (Figure S1C and Table S8, Supporting Information), which is consistent with the clinical finding that BCSCs are enriched in basal‐like breast cancers.^[^ ^34^ ^]^ Analysis of 1979 breast cancer patients in Molecular Taxonomy of Breast Cancer International Consortium (METABRIC) database demonstrated that patients that had cancer metastasis within 5 years had higher BCSC P‐Sig and lower BCSC N‐Sig expression in their primary tumor compared with those who did not have metastasis within 5 years (Figure 1D; Table S9, Supporting Information). We also validated the BCSC signature in multiple breast cancer cell lines, and found that BCSC P‐Sig expression was higher, while BCSC N‐Sig expression was lower, in breast cancer cells cultured as mammospheres, which are enriched for BCSCs, compared with those cultured on standard polystyrene tissue culture plates (Figure 1E).

### 2.2 Chemotherapy Induces BCSC Enrichment

Using the mRNA‐based BCSC signature, we next systemically evaluated how chemotherapeutic drugs affected the BCSC population. We injected human breast cancer cell line MDA‐MB‐231 into the mammary fat pad (MFP) of female severe combined immunodeficiency (SCID) mice, and treated the mice with paclitaxel. Paclitaxel treatment significantly increased the percentage of cells with aldehyde dehydrogenase activity (ALDH^+^) (Figure 1F,G) and the number of mammosphere‐forming cells (Figure 1H), both of which are indicators of BCSCs. RNA‐seq analysis revealed that paclitaxel treatment increased expression of the majority (80 out of 81) of BCSC P‐Sig genes and decreased expression of most (44 out of 67 detected) of BCSC N‐Sig genes (Figure 1I). Paclitaxel treatment also increased the percentage of ALDH^+^ cells (Figure S1D,E, Supporting Information) and the number of mammosphere‐forming cells (Figure S1F, Supporting Information) in multiple breast cancer cell lines. We also treated breast cancer cells with various FDA‐approved chemotherapeutic drugs paclitaxel, gemcitabine, and carboplatin, and all of these drugs increased BCSC P‐Sig and decreased BCSC N‐Sig expression (Figure 1J). Patient samples from METABRIC demonstrated that patients who received neoadjuvant chemotherapy had higher BCSC P‐Sig and lower BCSC N‐Sig expression in their primary tumor tissues compared with those patients who did not receive chemotherapy (Figure 1K; Table S10, Supporting Information). Taken together, we develop an mRNA‐based BCSC signature, validate its performance in breast cancer cell lines and primary patient tumor samples, and systemically demonstrate that chemotherapy increases breast cancer stemness in vitro and in vivo.

### 2.3 Chemotherapy Promotes BCSC Enrichment Through Induction of Polyamine Anabolism

**Figure 2:**
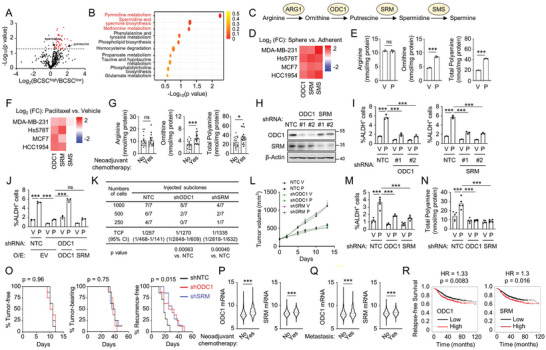
Chemotherapy induces BCSC enrichment through activation of polyamine anabolism. A,B) Metabolites information from 23 breast cancer patients in TCGA BRCA was compared between BCSC^high^ and BCSC^low^ groups (A), and metabolite ontology enrichment analysis was performed (B). C) Scheme of polyamine anabolic pathway. D) Adherent or sphere breast cancer cells were cultured and qPCR assay was performed. E) MDA‐MB‐231 cells were treated with vehicle (V) or 10 nM paclitaxel (P) for 3 days and polyamine metabolites were measured (mean ± SEM; n = 3). F) Breast cancer cells were treated with V or P for 3 days and qPCR assay was performed. G) Polyamine metabolites from primary breast cancer patient samples collected at Qilu Hospital of Shandong University were measured and compared between patients who received neoadjuvant chemotherapy (Yes) and patients who did not (No) (mean ± SEM; n = 17 in each group). H) Immunoblot assay was performed in MDA‐MB‐231 subclones for ODC1/SRM knockdown. I,J) MDA‐MB‐231 subclones were treated with V or P for 3 days, and ALDH assays were performed (mean ± SEM; n = 3). K) SCID mice were injected with indicated numbers of MDA‐MB‐231 NTC or ODC1/SRM knockdown subclone cells and tumor‐initiating cell frequency (TCF) with 95% confidence intervals (CI) was calculated 70 days after tumor injection. L‐N) 2 × 10^6^ MDA‐MB‐231 subclone cells were implanted into SCID mice. When tumor volume reached 200 mm^3^ (day 0), mice were treated with V or P (10 mg kg^−1^, days 0, 5, and 10), and tumor volumes were measured every 2–3 days (L). Tumors were harvested on day 13, and the percentage of ALDH^+^ cells (M) and polyamine levels in tumor tissue (N) were measured (mean ± SEM; n = 5). O) 2 × 10^6^ MDA‐MB‐231 subclone cells were implanted into SCID mice. When tumors were palpable, mice were treated with 10 mg kg^−1^ paclitaxel every 5 days until tumors were no longer palpable. Kaplan–Meier survival curves of tumor‐free (left), tumor‐bearing (center), and recurrence‐free (right) were plotted (n = 8). P,Q) mRNA expression of ODC1 and SRM in primary tumor tissue from patients who received neoadjuvant chemotherapy and who did not receive chemotherapy (P), and from patients who had metastasis within 5 years and who did not have metastasis within 5 years (Q), in METABRIC, was compared. R) Kaplan–Meier analysis of relapse‐free survival was performed based on ODC1 or SRM mRNA levels in patient that received chemotherapy (n = 1372). ^*^ *p* < 0.05, ^***^ *p* < 0.001; ns, not significant.

Next, we investigated the mechanism underlying chemotherapy‐induced BCSC enrichment in the perspective of cell metabolism. We stratified 21 patients from TCGA BRCA to BCSC^high^ and BCSC^low^ groups by the expression of the BCSC signature, and compared levels of 399 metabolites between the two groups (**Figure** **2**A; Table S11, Supporting Information). Metabolite ontology enrichment analysis revealed that the differential levels of metabolites were enriched in polyamine anabolism related pathways (Figure 2B). The key enzymes of polyamine anabolic pathway (Figure 2C), including ornithine decarboxylase 1 (ODC1), spermidine synthase (SRM), and spermine synthase (SMS), had higher expression in the BCSC^high^ group as compared to the BCSC^low^ group (Figure S2A, Supporting Information), and had higher expression in non‐adherent mammosphere cultures as compared to monolayer cultures in breast cancer cell lines (Figure 2D), suggesting a role of polyamine anabolism in the regulation of the BCSC phenotype.

Paclitaxel treatment elevated intracellular levels of ornithine and total polyamine (including putrescine, spermidine, and spermine) (Figure 2E; Figure S2B, Supporting Information), and increased expression of ODC1 and SRM (but not SMS) (Figure 2F) in breast cancer cell lines. We also observed increased ornithine and polyamine levels (Figure S2D, Supporting Information), and increased BCSC population (Figure S2E–H, Supporting Information), in a paclitaxel‐resistant MDA‐MB‐231 subclone we generated (Figure S2C, Supporting Information), compared to its sensitive counterpart. In addition, we collected 34 breast cancer patient samples (17 patients who received neoadjuvant chemotherapy, 17 patients who did not receive chemotherapy) from Qilu Hospital of Shandong University, and found significant increased intracellular levels of ornithine and polyamine in patients who received neoadjuvant chemotherapy (Figure 2G).

To investigate the regulatory role of polyamine anabolism in chemotherapy‐induced BCSC enrichment, we generated shRNA‐mediated non‐targeting control (NTC), ODC1, or SRM knockdown subclones of breast cancer cells (Figure 2H; Figure S2I, Supporting Information), and found that knockdown of ODC1 or SRM abrogated paclitaxel‐induced increases of ALDH^+^ cell population (Figure 2I; Figure S2J, Supporting Information), and decreased BCSC P‐Sig expression and increased BCSC N‐Sig expression (Figure S2K, Supporting Information). The effect of ODC1 knockdown in blocking paclitaxel‐induced BCSC enrichment was recused by overexpression of an shRNA‐resistant ODC1, but not by overexpression of SRM (Figure 2J), and was rescued by supplementation of spermidine in the cell culture medium (Figure S2L, Supporting Information). Co‐administration of difluoromethylornithine (DFMO), a specific inhibitor for ODC1^[^ ^35^ ^]^ also significantly blocked paclitaxel‐induced enrichment of ALDH^+^ cells in breast cancer cells (Figure S2M, Supporting Information). We also performed in vivo tumorigenicity assay by injecting 1000, 500, or 250 MDA‐MB‐231 NTC or ODC1/SRM knockdown subclone cells into SCID mice, and found that ODC1/SRM knockdown significantly decreased tumor‐initiating capacity (Figure 2K).

To investigate the regulatory role of ODC1/SRM on chemotherapy responses in vivo, we injected 2 × 10^6^ MDA‐MB‐231 NTC or ODC1/SRM knockdown subclones cells into SCID mice. Upon reaching a tumor volume of 200 mm^3^, mice were treated with paclitaxel. ODC1/SRM knockdown, which decreased polyamine levels in the tumor tissue (Figure 2N), did not affect primary tumor growth rate or paclitaxel sensitivity (Figure 2L), but attenuated paclitaxel‐induced enrichment of ALDH^+^ cells (Figure 2M), indicating that genetic inhibition of polyamine anabolism successfully blocks chemotherapy‐induced BCSC enrichment. We also performed tumor eradication and recurrence assay by implanting MDA‐MB‐231 NTC or ODC1/SRM knockdown subclone cells into SCID mice, treating the mice with paclitaxel when tumors became palpable, terminating treatment when tumors were fully eradicated, and monitoring tumor recurrence. ODC1/SRM knockdown did not alter time for tumor formation (Figure 2O, left panel) or tumor eradication (Figure 2O, middle panel), but significantly increased time for tumor recurrence (Figure 2O, right panel), indicating that ODC1/SRM knockdown decreases numbers of BCSCs in response to paclitaxel treatment, which is a major cause of tumor relapse.

To determine the clinical relevance of ODC1 and SRM expression in breast cancer, we analyzed METABRIC database and found that ODC1 and SRM expression was higher in primary tumor samples of patients who received neoadjuvant chemotherapy compared with patients who did not receive chemotherapy (Figure 2P; Table S12, Supporting Information), and in samples of patients who had metastasis within 5 years compared with patients who did not have metastasis within 5 years (Figure 2Q; Table S13, Supporting Information). Microarray and clinical data from 1372 breast cancer patients who received chemotherapy revealed that mRNA levels of ODC1 or SRM above the median were associated with decreased RFS (Figure 2R). Taken together, these data demonstrate that enhanced polyamine anabolism promotes chemotherapy‐induced BCSC enrichment in vitro and in vivo.

### 2.4 HIF‐1 Promotes Polyamine Anabolism and BCSC Enrichment in Response to Chemotherapy

**Figure 3:**
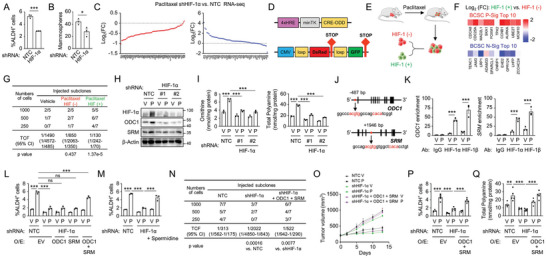
HIF‐1 promotes polyamine anabolism and BCSC enrichment in response to chemotherapy. A‐C) SCID mice injected with MDA‐MB‐231 NTC or HIF‐1*α* knockdown subclone cells were treated with paclitaxel, and tumors were harvested for ALDH (A; mean ± SEM; n = 3), mammosphere (B; mean ± SEM; n = 3), and RNA sequencing (C) assays. D) Scheme of HIF‐1 expression fluorescent tracking system. E‐G) SCID mice were injected with MDA‐MB‐231 cells transfected with HIF‐1 expression fluorescent tracking system and treated with paclitaxel. Tumor tissues were collected and sorted into DsRed^+^/GFP^−^ or GFP^+^ populations (E), and qPCR (F) and secondary limiting dilution transplantation (G) assay were performed. H,I) MDA‐MB‐231 subclones were treated with V or P for 3 days, and protein expression (H), and intracellular ornithine and polyamine levels (I; mean ± SEM; n = 3) was measured. J,K) MDA‐MB‐231 cells were treated with V or P and chromatin immunoprecipitation (ChIP) was performed with control IgG or antibody (Ab) against HIF‐1*α* or HIF‐1*β* (K; mean ± SEM; n = 3) and qPCR primers flanking the candidate HIF‐1 binding sites in the *ODC1* or *SRM* gene (J). L,M) MDA‐MB‐231 subclones were treated with V or P for 3 days in the absence or presence of spermidine, and ALDH assay was performed (mean ± SEM; n = 3). N) SCID mice were injected with indicated numbers of MDA‐MB‐231 subclone cells, and tumor‐initiating cell frequency (TCF) with 95% confidence intervals (CI) was calculated 70 days after tumor injection. O‐Q) 2 × 10^6^ MDA‐MB‐231 subclone cells were implanted in SCID mice. When tumor volume reached 200 mm^3^ (day 0), mice were treated with V or P (10 mg kg^−1^, days 0, 5, and 10), and tumor volumes were measured every 2–3 days (O). Tumors were harvested on day 13, and the percentage of ALDH^+^ cells (P) and polyamine levels in tumor tissues (Q) were measured (mean ± SEM; n = 5). ^*^ *p* < 0.05, ^**^ *p* < 0.01, ^***^ *p* < 0.001; ns, not significant.

We next investigated the molecular mechanism through which chemotherapy promotes polyamine anabolism in breast cancer. The expression of the BCSC P‐Sig and N‐Sig was correlated with HIF‐1‐regulated gene signature in TCGA BRCA dataset (Figure S3A, Supporting Information). Knockdown of HIF‐1*α* attenuated paclitaxel‐mediated increases of ALDH^+^ (**Figure** **3**A; Figure S3B, Supporting Information) and mammosphere‐forming (Figure 3B) cell population, and decreased expression of most BCSC P‐Sig genes and increased expression of most BCSC N‐Sig genes (Figure 3C), suggesting a critical role of HIF‐1 in the regulation of paclitaxel‐induced BCSC enrichment.

To address the role of HIF‐1 in the regulation of the BCSC phenotype, we transfected MDA‐MB‐231 with a HIF‐1 expression fluorescent tracking system^[^ ^36^ ^]^ (Figure 3D), implanted the cells into SCID mice, treated mice with paclitaxel, collected tumor tissues after treatment, and sorted cells into DsRed^+^/GFP^−^ (HIF‐1^−^) or GFP^+^ (HIF‐1^+^) populations (Figure 3E). The GFP^+^ population of MDA‐MB‐231 cells, which has high HIF‐1 expression and transcriptional activity, had increased BCSC P‐Sig gene expression and decreased BCSC N‐Sig expression, compared with DsRed^+^/GFP^−^ population (Figure 3F). We performed a secondary transplantation by injecting 1000, 500, or 250 sorted cells into SCID mice, and found that the GFP^+^ cells have significantly increased tumor‐initiating capacity compared with untreated cells or DsRed^+^/GFP^−^ cells (Figure 3G).

Next we investigated whether HIF‐1 promotes chemotherapy‐induced BCSC enrichment through regulation of polyamine anabolism. Analysis of TCGA BRCA dataset revealed a positive correlation of ODC1 and SRM expression with HIF‐1‐regulated gene signature (Figure S3C, Supporting Information). Knockdown of HIF‐1*α* blocked paclitaxel‐induced ODC1 and SRM expression (Figure 3H), and attenuated paclitaxel‐induced intracellular ornithine and polyamine levels (Figure 3I; Figure S3D, Supporting Information), indicating that paclitaxel promotes polyamine anabolism in a HIF‐1‐dependent manner. DNA sequences located in the 5′‐flanking region of *ODC1* gene and in the third intron of *SRM* gene (Figure 3J) were enriched by chromatin immunoprecipitation (ChIP) with antibody against HIF‐1*α* or HIF‐1*β*, when breast cancer cells were treated with paclitaxel for 3 days (Figure 3K; Figure S3C, Supporting Information), showing that *ODC1* and *SRM* are direct HIF‐1 target genes. Overexpression of both ODC1 and SRM (but not either of them alone), or supplementation of spermidine in the cell culture medium, reversed decreases of ALDH^+^ population mediated by HIF‐1*α* knockdown (Figure 3L,M), indicating that polyamine anabolism is downstream of HIF‐1 in the regulation of paclitaxel‐induced BCSC enrichment. Furthermore, HIF‐1*α* knockdown significantly decreased tumor‐initiating capacity in vivo, as measured by tumorigenicity assay, which was rescued by overexpression of ODC1 and SRM (Figure 3N). HIF‐1*α* knockdown in MDA‐MB‐231 cells markedly blocked paclitaxel‐induced enrichment of ALDH^+^ cells and polyamine levels, which were rescued by overexpression of ODC1 and SRM, in xenograft mice (Figure 3O–Q). Taken together, these data demonstrate that HIF‐1 promotes polyamine anabolism through transcriptional activation of *ODC1* and *SRM* genes, leading to chemotherapy‐induced BCSC enrichment.

### 2.5 A Novel HIF‐1 Inhibitor Britannin Decreases Polyamine Anabolism and Eradicates BCSCs

**Figure 4:**
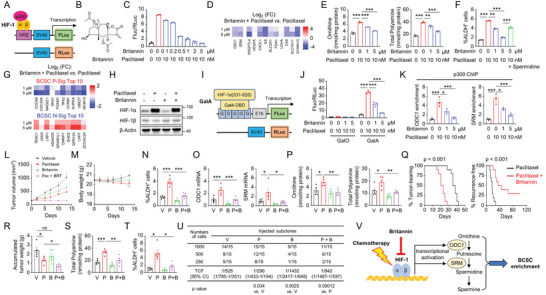
Britannin inhibits HIF‐1‐regulated polyamine anabolism and eradicates BCSCs. A) Scheme of HIF‐1 dual luciferase reporter system. B) Chemical structure of Britannin. C) SUM159 cells were transfected with HIF‐1 dual luciferase reporter system, treated as indicated for 3 days, and the FLuc/RLuc ratio was determined (mean ± SEM; n = 3). D‐G) MDA‐MB‐231 cells were treated with paclitaxel, alone or in combination with indicated doses of britannin, for 3 days. mRNA expression of HIF‐1‐target genes (D), levels of ornithine and polyamine (E; mean ± SEM; n = 3), the percentage of ALDH^+^ cells (F; mean ± SEM; n = 3), and mRNA expression of top 10 BCSC P‐Sig and N‐Sig genes (G) was determined. H) MDA‐MB‐231 cells were treated as indicated for 3 days and immunoblot assay was performed. I) Scheme of HIF‐1*α* TAD reporter system. J) MDA‐MB‐231 cells were transfected with HIF‐1*α* TAD function reporter system, treated as indicated for 3 days, and the Fluc/RLuc ratio was determined (mean ± SEM; n = 3). K) MDA‐MB‐231 cells were treated as indicated and ChIP assays were performed (mean ± SEM; n = 3). L‐P) 2 × 10^6^ MDA‐MB‐231 cells were implanted in SCID mice. When tumor volume reached 200 mm^3^ (day 0), mice were treated with vehicle, paclitaxel (P; 10 mg kg^−1^, days 0, 5, and 10), britannin (B; 5 mg kg^−1^, days 1–13), or the combination of paclitaxel and britannin (P + B). Tumor volumes (L) and mouse body weight (M) were measured every 2–3 days. Tumors were harvested on day 13, and the percentage of ALDH^+^ cells (N), mRNA expression of ODC1 and SRM (O), and levels of ornithine and polyamine in tumor tissues (P) were determined (mean ± SEM; n = 5). Q) 2 × 10^6^ MDA‐MB‐231 cells were implanted in SCID mice. When tumors were palpable, mice were treated with P or P + B, until tumors were no longer palpable. Kaplan–Meier survival curves of tumor‐bearing and recurrence‐free were plotted (n = 10). R‐U) MMTV‐PyMT mice were treated with V, P (5 mg kg^−1^, days 0, 5, and 10), B (5 mg kg^−1^, days 1–13), or P + B, when accumulated tumor volume of each mouse reached 150 mm^3^. Tumors were harvested on day 13, and the accumulated tumor weight (R), polyamine levels in tumor tissues (S), and the percentage of ALDH^+^ cells (T) were determined (mean ± SEM; n = 5). Tumor tissues were digested to single cells and secondary limiting dilution transplantation assay was performed in SCID mice (U). V) A proposed model of HIF‐1 in the regulation of polyamine anabolism and BCSC enrichment in response to chemotherapy. ^*^ *p* < 0.05, ^**^ *p* < 0.01, ^***^ *p* < 0.001; ns, not significant.

The critical role of HIF‐1‐controlled polyamine anabolic pathway in mediating chemotherapy‐induced BCSC enrichment indicates its potential as a target for effectively eliminating BCSCs. Natural products are a rich reservoir of bioactive compounds with therapeutic potentials against cancer. Therefore, we screened for novel HIF‐1 inhibitors in a natural product library that contains 4320 natural compounds by transfecting SUM159 cells with a HIF‐1 transcription activity reporter system^[^ ^37^ ^]^ (**Figure** **4**A) and treating the cells with paclitaxel, alone or in combination with each of the compounds in the library. We found britannin, a sesquiterpene lactone isolated from *Inula aucheriana* (Figure 4B), effectively inhibited HIF‐1 transcriptional activity in a dose‐dependent manner (Figure 4C). Compared with paclitaxel treatment alone, co‐administration of britannin decreased expression of ODC1 and SRM, as well as other classical HIF‐1 target genes (Figure 4D; Figure S4A, Supporting Information). Co‐administration of britannin dose‐dependently abrogated paclitaxel‐induced intracellular ornithine and polyamine levels (Figure 4E; Figure S4B, Supporting Information), and blocked paclitaxel‐induced enrichment of ALDH^+^ cells, which was rescued by supplementation of spermidine (Figure 4F; Figure S4C, Supporting Information). Co‐administration of britannin also decreased BCSC P‐Sig expression and increased BCSC N‐Sig expression (Figure 4G; Figure S4D, Supporting Information).

To illustrate the mechanism through which britannin inhibits HIF‐1 transcriptional activity, we treated MDA‐MB‐231 cells with paclitaxel, alone or in combination with britannin, and found that britannin did not decrease protein levels of HIF‐1*α* or HIF‐1*β* (Figure 4H) or decrease binding of HIF‐1*α* or HIF‐1*β* to the regulatory regions of *ODC1* or *SRM* genes (Figure S4E,F, Supporting Information). Thus, we hypothesized that britannin may inhibit transactivation domain (TAD) function of HIF‐1*α*. We co‐transfected MDA‐MB‐231 cells with a HIF‐1*α* TAD activity reporter system^[^ ^38^ ^]^ (Figure 4I), and found that co‐treatment of britannin attenuated paclitaxel‐induced HIF‐1*α* TAD activity in a dose‐dependent manner (Figure 4J). ChIP assay with HIF‐1 transcription co‐activator p300 demonstrated that britannin prevented paclitaxel‐induced p300 recruitment to the regulatory regions of *ODC1* and *SRM* genes that bind with HIF‐1 (Figure 4K), indicating that britannin inhibits HIF‐1 transcriptional activity through blocking recruitment of transcription co‐activator p300.

To evaluate the effect of britannin in vivo, we injected 2 × 10^6^ MDA‐MB‐231 cells into SCID mice and treated the mice with paclitaxel, alone or in combination with britannin. The combination treatment markedly suppressed primary tumor growth (Figure 4L), without affecting mouse appearance or body weight (Figure 4M). Co‐administration of britannin inhibited paclitaxel‐induced enrichment of ALDH^+^ cells (Figure 4N), decreased ODC1 and SRM expression (Figure 4O), as well as ornithine and polyamine levels (Figure 4P). We also performed tumor eradication and recurrence assay by treating the MDA‐MB‐231‐inoculated mice with paclitaxel, alone or in combination with britannin, when tumors were palpable. Co‐administration of britannin significantly shorten time for tumor eradication (Figure 4Q, left panel) and increased time for tumor recurrence (Figure 4Q, right panel). Notably, three out of ten mice in the combination‐treated group experienced no tumor recurrence 100 days after termination of drug treatment, suggesting a complete eradication of BCSCs when the mice were treated by combination of paclitaxel and britannin.

In addition, we employed a genetically engineered, autochthonous breast cancer model to further examine the effect of britannin in vivo. MMTV‐PyMT transgenic mice were treated by paclitaxel, alone or in combination with britannin, when accumulated tumor size reached 150 mm^3^. Co‐administration of britannin significantly decreased tumor weight (Figure 4R), polyamine levels (Figure 4S), and the percentage of ALDH^+^ cells (Figure 4T). We collected tumor tissues at the end point, digested them into single cells, and performed secondary transplantation in SCID mice. Paclitaxel treatment alone significantly increased tumor‐initiating capacity, which was blocked by co‐treatment of britannin (Figure 4U). Taken together, these data demonstrate a novel HIF‐1 inhibitor britannin effectively inhibits polyamine anabolism and prevents paclitaxel‐induced BCSC enrichment in vitro and in vivo.

## 3 Discussion

The response of breast cancer patients to chemotherapy is commonly evaluated based on RECIST (Response Evaluation Criteria in Solid Tumors), which majorly reflect tumor size.^[^ ^39^ ^]^ However, clinical response to chemotherapy in many breast cancer patients does not translate into a substantial improvement in overall survival.^[^ ^40^ ^]^ This paradox highlights the importance of tracking and eradicating BCSCs, the small but critical subpopulation of cancer cells that may drive breast cancer recurrence/metastasis, and finally patient mortality, in response to chemotherapy. Nevertheless, identification of BCSC population remains a big challenge, particularly in patient samples, due to lack of accurate markers. In the present study, we developed an mRNA expression‐based BCSC signature that can be used to quantitatively evaluate breast cancer stemness in patient samples. Using the BCSC signature, we systematically evaluated breast cancer cell responses to chemotherapy in the perspective of BCSCs in vitro and in vivo, elucidated the key role of polyamine anabolism in the regulation of BCSCs, and developed a novel HIF‐1 inhibitor britannin that may specifically eradicate chemotherapy‐induced BCSC enrichment (Figure 4V).

Polyamines are involved in a variety of biological processes including protein and nucleic acid synthesis, chromatin organization, cell proliferation and differentiation, apoptosis, and protection against oxidative damage.^[^ ^29^, ^41^ ^]^ Dysregulated polyamine metabolism is a common phenomenon in breast cancer.^[^ ^42^ ^]^ The requirement of elevated intracellular polyamine pools for tumor progression makes polyamine metabolic pathway an attracting target for breast cancer therapy. Several drugs targeting ODC1, including DFMO, PG‐11047, and SBP‐101 are under clinical trials for the treatment of breast cancer or other solid tumors.^[^ ^29^, ^41^ ^]^ It is noteworthy that our data have demonstrated that genetic or pharmacological inhibition of ODC1 significantly suppresses BCSC population, but does not affect primary tumor growth in animal experiments. Therefore, simple measurement of tumor sizes, which is largely determined by the bulk cell population, may not accurately reflect the efficacy of the drugs. Measurement of BCSC population is warranted to evaluate the efficacy of drugs that target polyamine metabolism pathway, and the BCSC signature we developed provides a valuable tool for the examination of BCSC population in patient samples. Although ODC1 and SRM were not listed in BCSC P‐Sig, they may have indirect but broad effects on the BCSC population through activation of polyamine metabolism. Therefore, the molecular mechanisms through which elevated polyamine anabolism regulates the expression of BCSC signature genes require further delineation in order to fully understand the regulatory role of ODC1 and SRM on BCSCs.

Recent evidence has suggested that polyamines play critical roles in remodeling the tumor immune microenvironment.^[^ ^29^, ^43^ ^]^ Elevated levels of polyamines promotes an immunosuppressive tumor microenvironment through inhibiting pro‐inflammatory M1 polarization and promoting anti‐inflammatory M2 polarization of macrophages.^[^ ^44^ ^]^ In addition, polyamines may establish a tumor‐permissive microenvironment through negatively regulating the functions of dendritic cells^[^ ^45^ ^]^ natural killer T cells^[^ ^46^ ^]^ CD8^+^ tumor infiltrating lymphocytes^[^ ^47^ ^]^ and Th1 cells^[^ ^48^ ^]^ and positively regulating the functions of regulatory T cells.^[^ ^49^ ^]^ One caveat of our study is that we focused on cancer cell intrinsic role of polyamine anabolism in the regulation of BCSC enrichment in response to chemotherapy, without addressing changes in the immune microenvironment that may also contribute to polyamine‐induced BCSC enrichment. Further in vitro and in vivo studies are warranted to determine the effect of elevated polyamine anabolism on the breast cancer immune microenvironment in response to chemotherapy, and the contribution of immune‐cancer cell interactions to chemotherapy‐induced BCSC enrichment.

The elevated polyamine level in breast cancer cells is a combinatorial effect of increased polyamine anabolism and decreased polyamine catabolism, both of which have been reported to be regulated by a series of transcription factors, including c‐MYC, JUN and c‐FOS.^[^ ^50^ ^]^ Here, we found that two key polyamine anabolic enzymes, ODC1 and SRM, are direct transcriptional targets of HIF‐1, the master regulator of cellular response to hypoxia (lack of oxygen).^[^ ^51^ ^]^ Hypoxic tumor microenvironment has been reported to promote polyamine metabolism^[^ ^52^ ^]^ and induces the BCSC phenotype.^[^ ^53^ ^]^ In our current study, chemotherapy induced HIF‐1 expression and promoted ODC1 and SRM expression at transcriptional level, leading to increased polyamine anabolism, independent of hypoxic tumor microenvironment. Our study further supports the feasibility of targeting HIF‐1 for the treatment of breast cancer in the setting of chemotherapy.

Our current study has demonstrated a HIF‐1‐controlled polyamine anabolic pathway that plays a critical role in the regulation of chemotherapy‐induced BCSC enrichment. HIF‐1 may regulate a series of genes that contribute to BCSC maintenance and specification under hypoxic microenvironment or in response to chemotherapy.^[^ ^54^ ^]^ Therefore, compared with targeting ODC1 or other polyamine metabolic enzymes, targeting HIF‐1 may have a broader effect in eradicating BCSCs. Using a high‐throughput luciferase reporter system for HIF‐1 transcriptional activity, we screened a library of 4320 natural compounds, and identified britannin as an efficient HIF‐1 inhibitor. Co‐administration of britannin can overcome the effect of chemotherapy on BCSC enrichment in xenograft and autochthonous mouse models. Further preclinical and clinical studies are warranted for safety profile, pharmacokinetics, and efficacy of britannin in breast cancer patients. Taken together, using an mRNA expression‐based BCSC signature, we demonstrate the critical role of polyamine anabolism in the regulation of chemotherapy‐induced BCSC enrichment, and provide compelling evidence that targeting HIF‐1‐controlled polyamine anabolism with novel inhibitor britannin efficiently eradicates BCSCs and may improve treatment outcome in breast cancer patients.

## 4 Experimental Section

### Development of BCSC Signature

mRNA expression of 1247 breast cancer patients was accessed from TCGA BRCA dataset, and machine learning based mRNA stemness index (mRNAsi) was accessed from previous publication.^[^ ^32^ ^]^ Pearson correlation of mRNA expression and mRNAsi in each patient was calculated. All genes with Pearson r > 0.70 were defined as BCSC P‐Sig, whereas Pearson r < −0.70 were defined as BCSC N‐Sig.

### Cell Culture and Reagents

MDA‐MB‐231, Hs578T, and MCF7 cells were maintained in DMEM; HCC1954 cells were maintained in RPMI‐1640, SUM159 cells were maintained in DMEM/F12, all of which were supplemented with 10% fetal bovine serum and 1% penicillin–streptomycin. Cells were maintained at 37 °C in a 5% CO_2_, 95% air incubator. All chemicals are listed in Table S1 (Supporting Information).

### Reverse Transcription and Quantitative PCR

Total RNA was extracted with TRIzol (ThermoFisher), precipitated with isopropanol, and treated with DNase (ThermoFisher). The concentration of RNA samples was measured with NanoDrop microvolume spectrophotometer (ThermoFisher), and the integrity of RNA samples was checked by agarose gel electrophoresis. cDNA was synthesized with HiFiScript cDNA Synthesis Kit (CWBio) and qPCR analysis was performed using SYBR Green (CWBio) and the CFX96 Touch real‐time PCR detection system (Bio‐Rad). The expression of each target mRNA relative to 18S rRNA was calculated based on the cycle threshold (Ct) as 2^−Δ(ΔCt)^, in which ΔCt = Ct (target) – Ct (18S rRNA), and Δ(ΔCt) = ΔCt (test sample) – ΔCt (control sample). Primer sequences (5′ to 3′) for RT‐qPCR are shown in Table S2 (Supporting Information).

### Immunoblot Assay

Cell lysates were prepared in RIPA buffer (Beyotime), separated by SDS‐PAGE, blotted onto nitrocellulose membranes, and probed with primary antibodies (Table S3, Supporting Information). The membranes were then probed with horseradish peroxidase–conjugated secondary antibodies (ZSGB‐Bio) and the chemiluminescent signal was detected using Immobilon Western Chemiluminescent HRP Substrate (EMD Millipore).

### Lentivirus Transduction

pLKO.1‐puro lentiviral vectors encoding shRNA targeting *HIF1A*, *ODC1*, and *SRM* were purchased from Sigma–Aldrich, and the clone IDs are shown in Table S4 (Supporting Information). Empty pLV‐C‐GFPSpark vector and pLV‐C‐GFPSpark vector encoding *ODC1* or *SRM* were purchased from Sino Biological. Lentiviruses were packaged in 293T cells and viral supernatant was collected 48 h after transfection. Breast cancer cells were transduced with viral supernatant supplemented with 10 µg mL^−1^ Polybrene (Solarbio). After 24 h, cells were replenished with fresh medium containing 0.5 µg mL^−1^ puromycin (Beyotime) and maintained in puromycin‐containing medium for selection of stably transfected clones.

### Aldehyde Dehydrogenase (ALDH) Assay

The ALDH assay was performed according to manufacturer's instructions (ALDEFLUOR Kit, Stem Cell Technologies). Cultured cells were trypsinized, whereas tumor tissues were minced, digested with 1 mg mL^−1^ of type 1 collagenase (Sigma‐Aldrich) at 37 °C for 30 min, and filtered through a 70‐µm cell strainer. 5 × 10^5^ cells were suspended in assay buffer containing 0.5 µM BODIPY‐animoacetaldehyde and incubated at 37 °C for 45 min. An aliquot of cells from each sample was treated with 50 mM diethylaminobenzaldehyde, an ALDH inhibitor, as a negative control for gating. Samples were analyzed by flow cytometry using FACScalibur (BD Biosciences).

### Mammosphere Assay

Breast cancer cells were trypsinized and filtered through a 70‐µm cell strainer. The number of live cells was determined using trypan blue staining and single cell suspensions were seeded in six‐well ultra‐low‐attachment plates (Corning) at a density of 5000 cells mL^−1^ in complete MammoCult Medium (Stem Cell Technologies). Mammosphere cultures were photographed 7 days later using a phase contrast microscope (Olympus) and mammospheres ≥ 50 µm in diameter were counted using ImageJ software.

### RNA Sequencing

MDA‐MB‐231 parental or knockdown subclones were injected into the MFP of SCID mice and treated as indicated for 13 days. Tumor tissues were collected and total RNA was isolated using TRIzol (ThermoFisher) and treated with DNase (ThermoFisher). Library preparation and sequencing using the DNBSEQ platform (MGI) were performed by BGI Genomics. RNA‐seq data were processed and analyzed using Dr. Tom online platform (http://report.bgi.com). Sequencing data was filtered with SOAPnuke and clean reads were mapped to the reference genome using Bowtie2. Expression level of gene was calculated by RSEM (v1.3.1) and differential expression analysis was performed using the DESeq2 (v1.4.5).

### Metabolite Ontology Analysis

mRNA expression and metabolites information of 23 breast cancer patients were accessed from previous publication.^[^ ^55^ ^]^ Patients were classified into BCSC^high^ and BCSC^low^ groups based on the expression of BCSC P‐Sig and N‐Sig genes. Differential levels of metabolites between two groups were compared and ontology‐based metabolite enrichment analysis was performed using MetaboAnalyst 5.0 (https://www.metaboanalyst.ca/).

### Ornithine and Total Polyamine Measurement

Levels of ornithine and total polyamine in cultured cells, tumor tissues, and serum were measured using ornithine assay kit (BioVision) and total polyamine assay kit (BioVision), respectively. For cultured cells and tumor tissues, 100 µL of ice‐cold ornithine or polyamine assay buffer was added to 1 × 10^6^ pelleted cells or 10 mg tissue samples. Samples were homogenized with a Dounce homogenizer, centrifuged at 10 000 g for 10 min at 4 °C, and supernatants were collected. For serum, samples were directly centrifuged at 10 000 g for 10 min at 4 °C to remove any insoluble precipitate. Supernatants from cultured cells, tumor tissues, or serum were transferred into a microcon‐10 kDa centrifugal filter (Millipore) and filtrates were collected by centrifugation at 10 000 g for 20 min at 4 °C. For ornithine measurement, resultant filtrates were mixed with a reaction mix including ornithine assay buffer, diluted ornithine converter mix, ornithine enzyme mix, ornithine developer mix, and diluted ornithine probe; for polyamine measurement, resultant filtrates were mixed with a reaction mix including polyamine assay buffer, polyamine enzyme mix, polyamine developer mix, and diluted polyamine probe. The mixture was incubated in a black 96‐well plate for 30 min at room temperature in dark and the fluorescence (Ex/Em = 535/587 nm) was measured using FLUOstar Omega microplate reader (BMG Labtech).

### Chromatin Immunoprecipitation (ChIP) Assay

Breast cancer cells were cross‐linked in 3.7% formaldehyde for 15 min, quenched in 0.125 M glycine for 5 min and lysed with SDS lysis buffer. Chromatin was sheared by sonication using Bioruptor (Diagenode) and incubated with antibodies (Table S5, Supporting Information) in the presence of Protein A/G magnetic beads (Selleck) for overnight. After sequential washing of the magnetic beads, DNA was eluted in 1% SDS/0.1 M NaHCO_3_, and crosslinks were reversed by the addition of 0.2 M NaCl. DNA was purified by phenol‐chloroform extraction and ethanol precipitation, and candidate binding sites were analyzed by qPCR. Primer sequences (5′ to 3′) are shown Table S6 (Supporting Information).

### HIF‐1 Expression Fluorescent Tracking System and Cell Sorting

The lentiviral vector encoding 4xHRE‐MinTK‐CRE‐ODD (Addgene, #141147) or CMV‐loxp‐DsRed‐loxp‐eGFP (Addgene, #141148) was co‐transfected with plasmid pR8.91 and pVSV‐G into 293T cells by using Polyjet (SignaGen). Viral supernatant from CMV‐loxp‐DsRed‐loxp‐eGFP transfected 293T cells was collected 48 h after transfection and was added to MDA‐MB‐231 cells with 10 µg mL^−1^ Polybrene (Solarbio) for overnight. Following selection, MDA‐MB‐231 cells were transduced with lentivirus encoding 4xHRE‐MinTK‐CRE‐ODD. MDA‐MB‐231 cells transfected with HIF‐1 expression fluorescent tracking system were then implanted into MFP of SCID mice and mice were treated with paclitaxel. Tumor tissues were collected at the end of treatment and were sorted into DsRed^+^/GFP^−^ or GFP^+^ populations.

### Luciferase Reporter Assay

For HIF‐1 inhibitor screening, SUM159 cells were stably transfected with reporter plasmid pHRE‐SV‐Firefly and control plasmid pSV‐Renilla using PolyJet (SignaGen). 1 × 10^4^ transfected SUM159 cells were seeded in each well of 96‐well plate and treated with paclitaxel, alone or in combination with each of 4320 natural compounds from Natural Product Library (TargetMol) for 3 days. The ratio of Firefly/Renilla luciferase activity was determined by using the Dual‐luciferase assay system (Promega) and Centro LB960 microplate luminometer (Berthold).

For HIF‐1*α* transactivation domain function examination, MDA‐MB‐231 cells were transfected with GalA or GalO, reporter plasmid pG5‐E1b‐Firefly, and control plasmid pSV‐Renilla using PolyJet (SignaGen). Cells were treated with paclitaxel, alone or in combination with 1 or 5 µM britannin, for 3 days. The ratio of Firefly/Renilla luciferase activity was determined by using the Dual‐luciferase assay system (Promega) and Centro LB960 microplate luminometer (Berthold).

### Animal experiment

Animal protocols were approved by Institutional Animal Care and Use Committee of Cheeloo College of Medicine, Shandong University (ECSBMSSDU2021‐2‐009 and ECSBMSSDU2021‐2‐117).

For drug treatment assays with SCID mice, 2 × 10^6^ MDA‐MB‐231 parental or knockdown subclone cells were injected into the MFP of 5‐to‐7‐week‐old female mice in a 1:1 suspension of Matrigel (Corning) in PBS. When tumor volume reached 200 mm^3^ (day 0), mice were grouped randomly and treated as indicated. Tumor volumes and mouse body weight were measured every 2–3 days, and tumors were harvested on day 13 for different assays. For drug treatment assays with MMTV‐PyMT transgenic mice, mice were treated when the accumulated volumes of mammary tumors in each mouse reached 150 mm^3^.

For tumor eradication and recurrence assays, 2 × 10^6^ MDA‐MB‐231 parental or knockdown subclone cells were injected into the MFP of 5‐to‐7‐week‐old female mice in a 1:1 suspension of Matrigel in PBS. When tumors became palpable, mice were treated as indicated until tumors were no longer palpable. Kaplan–Meier survival curves of tumor‐free, tumor‐bearing, and recurrence‐free were plotted and p values of log‐rank tests were calculated.

For tumorigenicity assays, 1000, 500, or 250 cells of MDA‐MB‐231 knockdown subclone or cells from tumor tissues in treated mice were injected into MFP of 5‐to‐7‐week‐old female SCID mice in a 1:1 suspension of Matrigel in PBS. Seventy days after injection, the number of mice that developed palpable tumors was recorded. The tumor‐initiating cell frequency (TCF) with 95% confidence intervals (CI) and p value was calculated using ELDA software (https://bioinf.wehi.edu.au/software/elda/).

For all assays above, primary tumors were measured for length (L) and width (W), and tumor volume (V) was calculated as V = L × W^2^ × 0.524. Paclitaxel and britannin were given by intraperitoneal injection. Paclitaxel was given every 5 days at the dose of 10 mg kg^−1^ for SCID mice and 5 mg kg^−1^ for MMTV‐PyMT mice, britannin was given daily at the dose of 5 mg kg^−1^.

### Human Specimens

Human specimen study protocols and sample use were approved by Medical Ethics Review Committee of Cheeloo College of Medicine, Shandong University (ECSBMSSDU2022‐1‐58). All clinical samples were analyzed in a de‐identified fashion. All experiments were carried out in conformity to the principles set out in the WMA Declaration of Helsinki. Informed written consent for possible scientific research was provided by all patients before the surgery operation begins.

Surgically resected, liquid nitrogen frozen breast cancer tissue samples were collected retrospectively from the Department of Breast Surgery, Qilu Hospital of Shandong University. A total of 34 primary tumor samples were collected, with 17 patients who received neoadjuvant chemotherapy before tumor tissues were resected and 17 patients who did not receive neoadjuvant chemotherapy.

### Bioinformatics

mRNA expression of 1247 breast cancer patients was accessed from TCGA BRCA dataset, and was compared between cancer and normal breast tissue, or among different subtypes of breast cancer. mRNA levels of an established BCSC‐signature^[^ ^33^ ^]^ and HIF‐signature^[^ ^9^ ^]^ were accessed from TCGA BRCA dataset, and Pearson correlation tests were performed. Kaplan–Meier curves were generated from a dataset containing gene expression and survival data from 2032 breast cancer patients, who were stratified by BCSC P‐Sig and N‐Sig expression in primary tumor; or a dataset from 1372 breast cancer patients that received chemotherapy, who were stratified by ODC1 or SRM expression.

### Statistics

All data were expressed as mean ± SEM. Comparisons were performed by Student's t test between two groups and by One‐way ANOVA with post‐test among three or more groups, unless otherwise specified. For all tests, p values < 0.05 were considered significant.

## Conflict of Interest

The authors declare no conflict of interest.

## Author Contributions

G.J., J.L., Z.Z., and J.L. contributed equally to this work. G.J., J.L., Z.Z., J.L., K.Z., Q.M., R.S., and H.L. designed the research study. G.J., J.L., Z.Z., J.L., Y.Y., Z.W., H.F., K.J., X.J., H.X., G.W., Y.Z., X.D., Y.Z., Y.W., Y.Y., Z.L., and H.L. performed the experiments and acquired data, J.L., Y.Y., Z.Z., X.J., H.X., Z.L., K.Z., and Q.M. performed statistical analyses. Z.Z. and K.J. performed database analyses. G.J., J.L., Z.Z., J.L., Y.Y., Z.W., H.F., K.J., H.X., G.W., Y.Z., X.D. Y.Z., Y.Y., Z.L., K.Z., R.S., and H.L. analyzed the data, G.J., J.L., and H.L. wrote the manuscript. K.Z., Q.M., R.S., and H.L. supervised the study.

## Supporting information

Supporting Information

Supporting Information

Supporting Tables

## Data Availability

The data that support the findings of this study are available from the corresponding author upon reasonable request.
