# Supplementary material for: Polyamine Anabolism Promotes Chemotherapy‐Induced Breast Cancer Stem Cell Enrichment
Source: Adv Sci (Weinh). 2024 Jul 26;11(40):2404853. doi: 10.1002/advs.202404853 (PMC11516096; doi:10.1002/advs.202404853)
Supplement: Supplementary file 1 — Supporting Information [file ADVS-11-2404853-s003.docx]

**Supplementary information for**

**Polyamine anabolism promotes chemotherapy-induced breast cancer stem cell enrichment**

Guangyu Ji, Jia Liu, Zhiqun Zhao, Jie Lan, You Yang, Zheng Wang, Huijing Feng, Kai Ji, Xiaofeng Jiang, Huize Xia, Guangyao Wei, Yajing Zhang, Yuhong Zhang, Xinlong Du, Yawen Wang, Yuanyuan Yang, Zhaojian Liu, Kai Zhang, Qi Mei, Rong Sun, and Haiquan Lu

This file includes:

Supplementary figure legends for figures S1-S4;

Supplementary tables S1-S6;

Supplementary tables S7-S13 (in separate excel files).

**Supplementary figure legends**

**Figure S1. An mRNA expression-based BCSC signature demonstrates chemotherapy-induced BCSC enrichment.**

**(A**) Pearson correlation of BCSC P-Sig and N-Sig with BCSC signature from literature in TCGA BRCA samples was calculated. **(B and C)** BCSC P-Sig and N-Sig expression in cancer versus normal tissue (B) and in different subtypes of breast cancer (C) in TCGA BRCA was compared. ***p < 0.001 vs. normal in (B); ***p < 0.001 vs. all other subtypes in (C). **(D-F)** Breast cancer cells were treated with vehicle (V) or paclitaxel (P) for 3 days, and ALDH (D, E) and mammosphere (F) assays were performed (mean ± SEM; n = 3). *p < 0.05, **p < 0.01, ***p < 0.001 vs. V.

**Figure S2. Chemotherapy induces BCSC enrichment through activation of polyamine anabolism.**

**(A**) mRNA expression of key polyamine anabolic genes in patients from TCGA BRCA was compared between BCSC^high^ and BCSC^low^ groups. ***p < 0.001. **(B)** Breast cancer cells were treated with vehicle (V) or paclitaxel (P) for 3 days and levels of arginine, ornithine, and total polyamine in the cells were determined (mean ± SEM; n = 3). ***p < 0.001 vs. V; ns, not significant. **(C)** MDA-MB-231 cells sensitive (S) or resistant (R) to paclitaxel were treated with indicated doses of paclitaxel and CCK-8 assay was performed. **(D)** Levels of arginine, ornithine, and total polyamine in MDA-MB-231 cells sensitive or resistant to paclitaxel were determined (mean ± SEM; n = 3). **p < 0.01, ***p < 0.001 vs. S; ns, not significant. **(E)** qPCR assay was performed for the mRNA expression of top 10 BCSC P-Sig and N-Sig genes in MDA-MB-231 cells sensitive or resistant to paclitaxel. **(F-H)** The percentage of ALDH^+^ cells (F, G) and the number of mammosphere-forming cells (H) was determined in MDA-MB-231 cells sensitive or resistant to paclitaxel (mean ± SEM; n = 3). *p < 0.05, ***p < 0.001 vs. S. **(I)** qPCR assay was performed in breast cancer cell line NTC or ODC1/SRM knockdown subclones. ***p < 0.001 vs. NTC. **(J)** Breast cancer cell line NTC or ODC1/SRM knockdown subclones were treated with V or P for 3 days, and ALDH assays were performed (mean ± SEM; n = 3). *p < 0.05, **p < 0.01, ***p < 0.001 vs. NTC-V; ^###^p < 0.001 vs. NTC-P. **(K)** MDA-MB-231 NTC or ODC1/SRM knockdown subclones were treated with V or P for 3 days, and qPCR assay was performed. **(L)** MDA-MB-231 subclones were treated with V or P for 3 days in the absence or presence of spermidine, and ALDH assay was performed (mean ± SEM; n = 3). ***p < 0.001 vs. NTC-V; ^###^p < 0.001 vs. NTC-P; ^&&&^p < 0.001 vs. shODC1/SRM-P. **(M)** Breast cancer cells were treated with paclitaxel, alone or in combination with difluoromethylornithine (D), for 3 days, and ALDH assays were performed (mean ± SEM; n = 3). *p < 0.05, ***p < 0.001 vs. V; ^###^p < 0.001 vs. P; ns, not significant.

**Figure S3.** **HIF-1 promotes polyamine anabolism and BCSC enrichment in response to chemotherapy.**

**(A)** Pearson correlation of BCSC P-Sig and N-Sig with HIF signature in TCGA BRCA samples was calculated. **(B)** SCID mice injected with MDA-MB-231 NTC or HIF-1α knockdown subclone cells were treated with paclitaxel, and tumors were harvested for ALDH assay (mean ± SEM; n = 3), and a representative result from flow cytometry is shown. **(C)** Pearson correlation of ODC1 and SRM mRNA expression with HIF signature in TCGA BRCA samples was calculated. **(D)** Breast cancer cell line NTC or HIF-1α knockdown subclones were treated with V or P for 3 days, and intracellular ornithine and polyamine levels (I) was measured (mean ± SEM; n = 3). *p < 0.05, **p < 0.01, ***p < 0.001 vs. NTC-V; ^###^p < 0.001 vs. NTC-P. **(E)** Breast cancer cells were treated with V or P and chromatin immunoprecipitation (ChIP) was performed with control IgG or antibody (Ab) against HIF-1α or HIF-1β (mean ± SEM; n = 3). ***p < 0.001 vs. corresponding V.

**Figure S4. Britannin inhibits HIF-1-regulated polyamine anabolism and eradicates BCSCs.**

**(A-D)** Breast cancer cells were treated with paclitaxel, alone or in combination with indicated doses of britannin, for 3 days. mRNA expression of HIF-1-target genes (A), levels of ornithine and polyamine (B), the percentage of ALDH^+^ cells (C), and mRNA expression of top 10 BCSC P-Sig and N-Sig genes (D) was determined (mean ± SEM; n = 3). ***p < 0.001 vs. paclitaxel-0 nM-britannin-0 μM; ^#^p < 0.05, ^##^p < 0.01, ^###^p < 0.001 vs. paclitaxel-10 nM-britannin-0 μM; ^&&&^p < 0.001 vs. paclitaxel-10 nM-britannin-5 μM. **(E and F)** MDA-MB-231 cells were treated with paclitaxel, alone or in combination with indicated doses of britannin, for 3 days. ChIP assays were performed with antibody against HIF-1α (E) or HIF-1β (F) followed by qPCR using primers flanking HIF-1 binding sites in *ODC1* and *SRM* genes (mean ± SEM; n = 3). ***p < 0.001 vs. vehicle; ns, not significant.

**Supplementary tables S1-S6**

**Table S1. Chemical information.**

| **Chemical** | **Manufacture** | **Catalog #** |
| --- | --- | --- |
| Paclitaxel | Sigma-Aldrich | T7402 |
| Gemcitabine | Sigma-Aldrich | G6423 |
| Carboplatin | Sigma-Aldrich | C2538 |
| Spermidine | Sigma-Aldrich | S0266 |
| Difluoromethylornithine | Santa Cruz | sc-204723 |
| Britannin | Topscience | T5793 |
| TRIzol | Invitrogen | 15596026 |
| Isopropanol | Sinopharm Chemical | 80109218 |
| DNase | Thermo Fisher | EN0521 |
| SYBR Green | CWBio | CW0957 |
| RIPA buffer | Beyotime | P0013 |
| Immobilon Western Chemiluminescent HRP Substrate | Millipore | WBKLS0500 |
| Polyjet | Signagen | SL100688 |
| Polybrene | Solarbio | H8761 |
| Puromycin | Beyotime | ST551 |
| Matrigel | Corning | 356234 |
| Type I collagenase | Sigma-Aldrich | C0130 |

**Table S2. Oligonucleotide sequence of RT-qPCR primers.**

| **Gene** | **sequence (5' to 3')** | **Primer Efficiency (%)** |
| --- | --- | --- |
| *CDCA8* | Forward: CTTCGCCCTTGGAGGAAACAA | 106.9 |
|  | Reverse: GGTGTCTGAATAGCTTCTGCTG |  |
| *CDC25A* | Forward: GTGAAGGCGCTATTTGGCG | 109.4 |
|  | Reverse: TGGTTGCTCATAATCACTGCC |  |
| *MAD2L1* | Forward: GGACTCACCTTGCTTGTAACTAC | 99.5 |
|  | Reverse: GATCACTGAACGGATTTCATCCT |  |
| *SKA1* | Forward: CCTGAACCCGTAAAGAAGCCT | 99.4 |
|  | Reverse: TCATGTACGAAGGAACACCATTG |  |
| *FOXM1* | Forward: CGTCGGCCACTGATTCTCAAA | 90.8 |
|  | Reverse: GGCAGGGGATCTCTTAGGTTC |  |
| *TPX2* | Forward: ATGGAACTGGAGGGCTTTTTC | 90.6 |
|  | Reverse: TGTTGTCAACTGGTTTCAAAGGT |  |
| *CCNB1* | Forward: AATAAGGCGAAGATCAACATGGC | 93.7 |
|  | Reverse: TTTGTTACCAATGTCCCCAAGAG |  |
| *AURKA* | Forward: CAGACTGGATACCGGGACC | 107.1 |
|  | Reverse: CTTCAGCACGTTTTTGCACTG |  |
| *UBE2T* | Forward: ATCCCTCAACATCGCAACTGT | 106.8 |
|  | Reverse: CAGCCTCTGGTAGATTATCAAGC |  |
| *MCM10* | Forward: CCCCTACAGACGATTTCTCGG | 80.6 |
|  | Reverse: CAGATGGGTTGAGTCGTTTCC |  |
| *TENC1* | Forward: CTTAGACCTCACCTACGTGACG | 109.8 |
|  | Reverse: TTGTCCCGGTGCTTGGATTG |  |
| *JAM3* | Forward: CGGCTGCCTGACTTCTTCC | 85.5 |
|  | Reverse: TGGGGTTCGATTGCTGGATTT |  |
| *LRP1* | Forward: CTATCGACGCCCCTAAGACTT | 102.2 |
|  | Reverse: CATCGCTGGGCCTTACTCT |  |
| *ADAM33* | Forward: CCAACCACACGGATCATTGC | 84.2 |
|  | Reverse: ACGCAGATAATAGCTGGCATTC |  |
| *SPARCL1* | Forward: ACGGTAGCACCTGACAACAC | 108.7 |
|  | Reverse: ATGGTGGGAATCGTCTTCTGT |  |
| *CNRIP1* | Forward: TAATGACGGCCCGGTCTTTTA | 93.6 |
|  | Reverse: TGCAGCGTGCTGGGTTTAAT |  |
| *EHD2* | Forward: TCCGCAAACTCAACCCTTTC | 90.7 |
|  | Reverse: TCTCCAGGACCTGATTAGGGA |  |
| *GPR124* | Forward: CCCTACGCCAAGTGGTGTTC | 107.9 |
|  | Reverse: GAAGGTGCAGTCGTGGATGAG |  |
| *LHFP* | Forward: CTCCTGCGTGGGGTTCTTTAT | 94.1 |
|  | Reverse: CCGGTCACTATGGTGCAGAT |  |
| *ZCCHC24* | Forward: CGAGGGCCTGACTCCATAC | 109.1 |
|  | Reverse: ACACGTTGATGTGGCACTTGA |  |
| *ODC1* | Forward: TTTACTGCCAAGGACATTCTGG | 91.2 |
|  | Reverse: GGAGAGCTTTTAACCACCTCAG |  |
| *SRM* | Forward: GTGGTGGCCTATGCCTACTG | 105.1 |
|  | Reverse: CTCCTGGAAGTTCGTGCTCG |  |
| *SMS* | Forward: TGGGCGGGTGAAACGATTAC | 89.1 |
|  | Reverse: CCAAACTGCTTCGAGTGTAGAA |  |
| *SMOX* | Forward: CGGATGACCCTCTCAGTCG | 94.7 |
|  | Reverse: GCGTGTCCAAGTTTCACACT |  |
| *ANGPTL4* | Forward: GGACACGGCCTATAGCCTG | 85.8 |
|  | Reverse: CTCTTGGCGCAGTTCTTGTC |  |
| *VEGFA* | Forward: AGGGCAGAATCATCACGAAGT | 96.2 |
|  | Reverse: AGGGTCTCGATTGGATGGCA |  |
| *CXCL1* | Forward: GCGCCCAAACCGAAGTCATA | 98.5 |
|  | Reverse: ATGGGGGATGCAGGATTGAG |  |
| *IL6* | Forward: ACTCACCTCTTCAGAACGAATTG | 95.3 |
|  | Reverse: CCATCTTTGGAAGGTTCAGGTTG |  |
| *SLC2A1* | Forward: GGCCAAGAGTGTGCTAAAGAA | 97.9 |
|  | Reverse: ACAGCGTTGATGCCAGACAG |  |
| *PDK1* | Forward: ACCAGGACAGCCAATACAAG | 109.7 |
|  | Reverse: CCTCGGTCACTCATCTTCAC |  |
| *LDHA* | Forward: ATCTTGACCTACGTGGCTTGGA | 104.3 |
|  | Reverse: CCATACAGGCACACTGGAATCTC |  |
| *CA9* | Forward: GGATCTACCTACTGTTGAGGCT | 97.1 |
|  | Reverse: CATAGCGCCAATGACTCTGGT |  |
| *SLC7A11* | Forward: AGGGTCACCTTCCAGAAATC | 94.6 |
|  | Reverse: GAAGATAAATCAGCCCAGCA |  |
| *S100A10* | Forward: GGCTACTTAACAAAGGAGGACC | 92.6 |
|  | Reverse: GAGGCCCGCAATTAGGGAAA |  |
| *18S* | Forward: CGGCGACGACCCATTCGAAC | 92.2 |
|  | Reverse: GAATCGAACCCTGATTCCCCGTC |  |

**Table S3. Primary antibody information for immunoblot assays.**

| **Antibody** | **Manufacture** | **Catalog #** |
| --- | --- | --- |
| HIF-1α | Cayman Chemical | 10006421 |
| HIF-1β | Novus | NB100-110 |
| ODC1 | Proteintech | 28728-1-AP |
| SRM | Proteintech | 19858-1-AP |
| Actin | Santa Cruz | sc-1616 |

**Table S4. Oligonucleotide information of shRNAs.**

| **shRNA** | **Clone ID** |
| --- | --- |
| ODC1 #1 | TRCN0000078435 |
| ODC1 #2 | TRCN0000078433 |
| SRM #1 | TRCN0000045730 |
| SRM #2 | TRCN0000290715 |
| HIF1A #1 | TRCN0000003808 |
| HIF1A #2 | TRCN0000010819 |

**Table S5. Primary antibody information for ChIP assays.**

| **Antibody** | **Manufacture** | **Catalog #** |
| --- | --- | --- |
| HIF-1α | Novus Biologicals | NB100-479 |
| HIF-1β | Novus Biologicals | NB100-110 |
| p300 | Novus Biologicals | NB100-616 |

**Table S6. Oligonucleotide sequence of ChIP primers.**

| *ODC1* | Forward: cctctgctccctagctggt |
| --- | --- |
|  | Reverse: gagagcggaaaagggaaatc |
| *SRM* | Forward: tgccctctaggagctgatgt |
|  | Reverse: aaatggggtttcaccaggtt |

**Supplementary tables S7-S13 (in separate excel files)**

Tables S7. Correlation of gene expression and mRNAsi in TCGA BRCA;

Tables S8. BCSC signature gene expression in Cancer vs Normal and in different subtypes of breast cancer in TCGA BRCA;

Tables S9. BCSC signature gene expression in metastatic vs non-metastatic cancer within 5 years in METABRIC database;

Tables S10. BCSC signature gene expression in chemotherapy vs non-chemotherapy patients in METABRIC database;

Tables S11. Levels of metabolites in BCSC^high^ and BCSC^low^ groups in TCGA BRCA;

Tables S12. ODC1 and SRM expression in chemotherapy vs non-chemotherapy patients in METABRIC database;

Tables S13. ODC1 and SRM expression in metastatic vs non-metastatic cancer within 5 years in METABRIC database.
