# Supplementary material for: Polyamine Anabolism Promotes Chemotherapy‐Induced Breast Cancer Stem Cell Enrichment
Source: Adv Sci (Weinh). 2024 Jul 26;11(40):2404853. doi: 10.1002/advs.202404853 (PMC11516096; doi:10.1002/advs.202404853)
Supplement: Supplementary file 2 — Supporting Information [file ADVS-11-2404853-s002.pdf]

ADVANCED SCIENCE
Open Access

Supporting Information

for *Adv. Sci.*, DOI 10.1002/advs.202404853

Polyamine Anabolism Promotes Chemotherapy-Induced Breast Cancer Stem Cell Enrichment

Guangyu Ji, Jia Liu, Zhiqun Zhao, Jie Lan, You Yang, Zheng Wang, Huijing Feng, Kai Ji, Xiaofeng Jiang, Huize Xia, Guangyao Wei, Yajing Zhang, Yuhong Zhang, Xinlong Du, Yawen Wang, Yuanyuan Yang, Zhaojian Liu, Kai Zhang, Qi Mei*, Rong Sun* and Haiquan Lu**

Figure S1

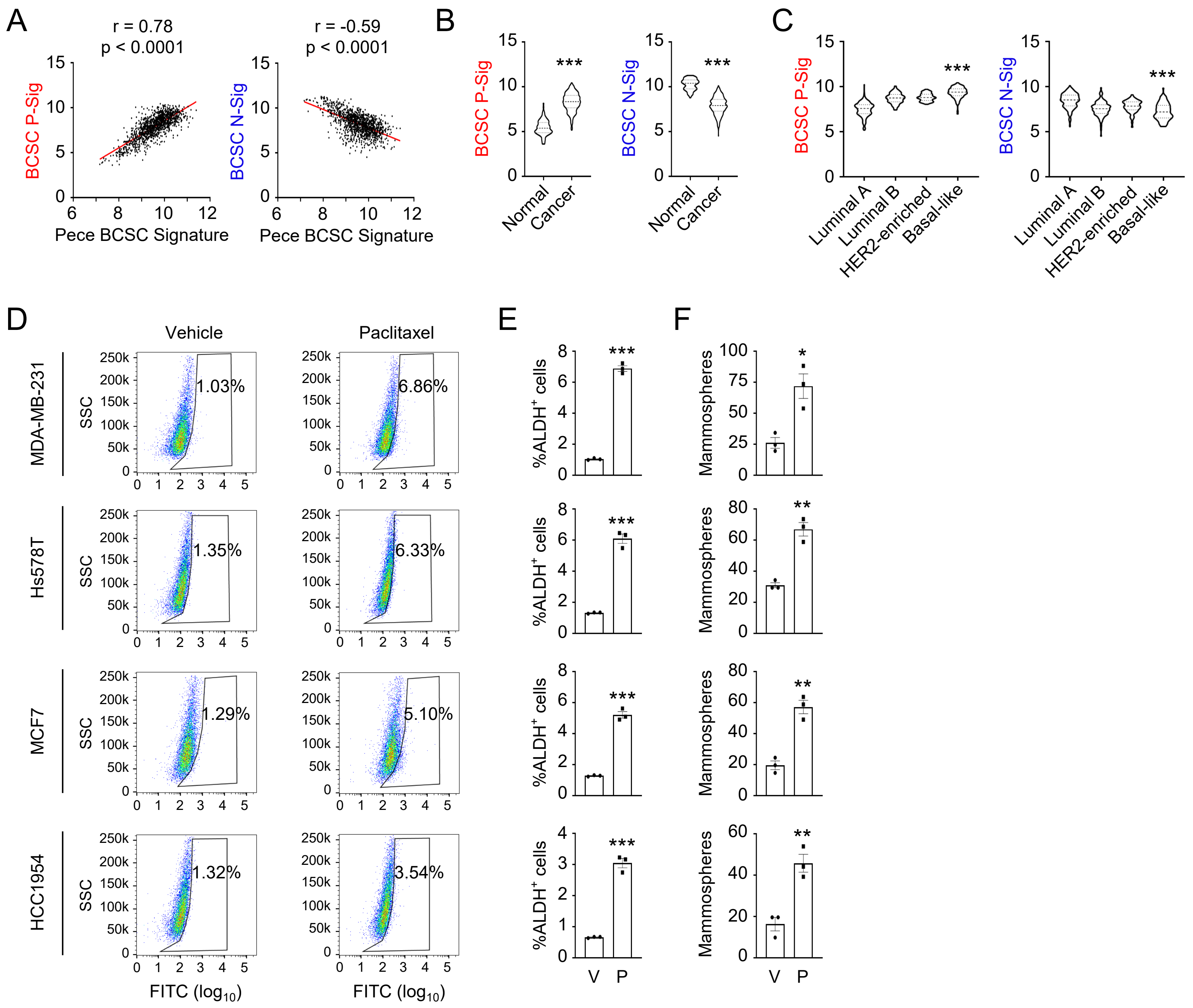
A
r = 0.78
p < 0.0001
BCSC P-Sig
Pece BCSC Signature
r = -0.59
p < 0.0001
BCSC N-Sig
Pece BCSC Signature
B
BCSC P-Sig
BCSC N-Sig
Normal
Cancer
***
C
BCSC P-Sig
BCSC N-Sig
Luminal A
Luminal B
HER2-enriched
Basal-like
***
D
Vehicle
Paclitaxel
MDA-MB-231
Hs578T
MCF7
HCC1954
SSC
1.03%
6.86%
1.35%
6.33%
1.29%
5.10%
1.32%
3.54%
FITC (log10)
E
%ALDH+ cells
V
P
F
Mammospheres
*
**

Figure S2

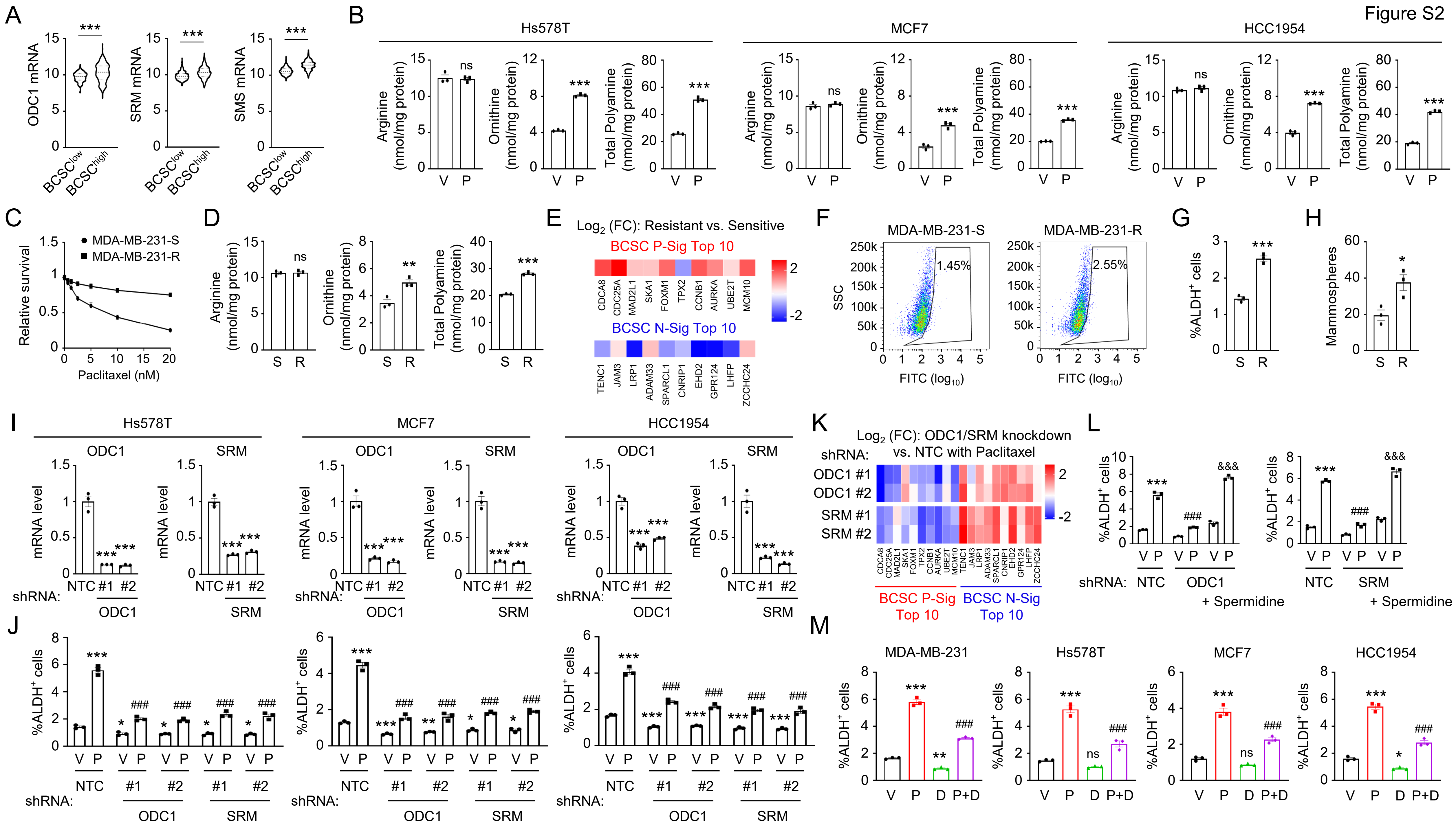

A
ODC1 mRNA
SRM mRNA
SMS mRNA
BCSC low
BCSC high
B
Hs578T
MCF7
HCC1954
Arginine (nmol/mg protein)
Ornithine (nmol/mg protein)
Total Polyamine (nmol/mg protein)
V P
C
MDA-MB-231-S
MDA-MB-231-R
Relative survival
Paclitaxel (nM)
D
S R
E
Log2 (FC): Resistant vs. Sensitive
BCSC P-Sig Top 10
CDCA8 CDC25A MAD2L1 SKA1 FOXM1 TPX2 CCNB1 AURKA UBE2T MCM10
BCSC N-Sig Top 10
TENC1 JAM3 LRP1 ADAM33 SPARCL1 CNRIP1 EHD2 GPR124 LHFP ZCCHC24
F
MDA-MB-231-S
MDA-MB-231-R
SSC
FITC (log10)
1.45%
2.55%
G
%ALDH+ cells
H
Mammospheres
I
ODC1
SRM
mRNA level
shRNA:
NTC #1 #2
J
%ALDH+ cells
K
Log2 (FC): ODC1/SRM knockdown vs. NTC with Paclitaxel
shRNA:
ODC1 #1
ODC1 #2
SRM #1
SRM #2
BCSC P-Sig Top 10
BCSC N-Sig Top 10
L
+ Spermidine
M
MDA-MB-231
V P D P+D

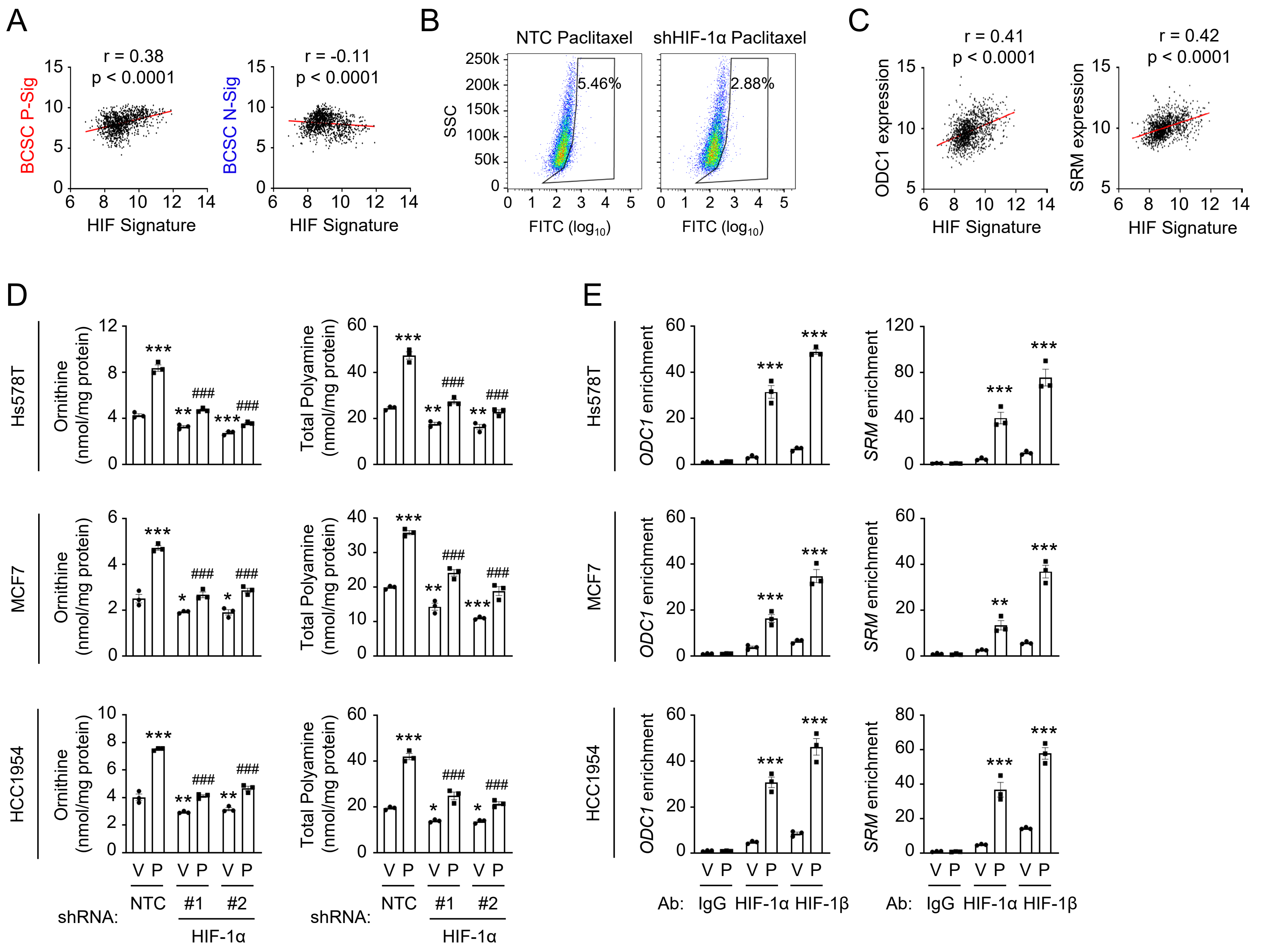
A
r = 0.38
p < 0.0001
BCSC P-Sig
HIF Signature
r = -0.11
p < 0.0001
BCSC N-Sig
HIF Signature
B
NTC Paclitaxel
shHIF-1α Paclitaxel
5.46%
2.88%
SSC
FITC (log10)
C
r = 0.41
p < 0.0001
ODC1 expression
HIF Signature
r = 0.42
p < 0.0001
SRM expression
HIF Signature
D
Hs578T
MCF7
HCC1954
Ornithine (nmol/mg protein)
Total Polyamine (nmol/mg protein)
V P V P V P
shRNA: NTC #1 #2
HIF-1α
E
ODC1 enrichment
SRM enrichment
Ab: IgG HIF-1α HIF-1β

Figure S4

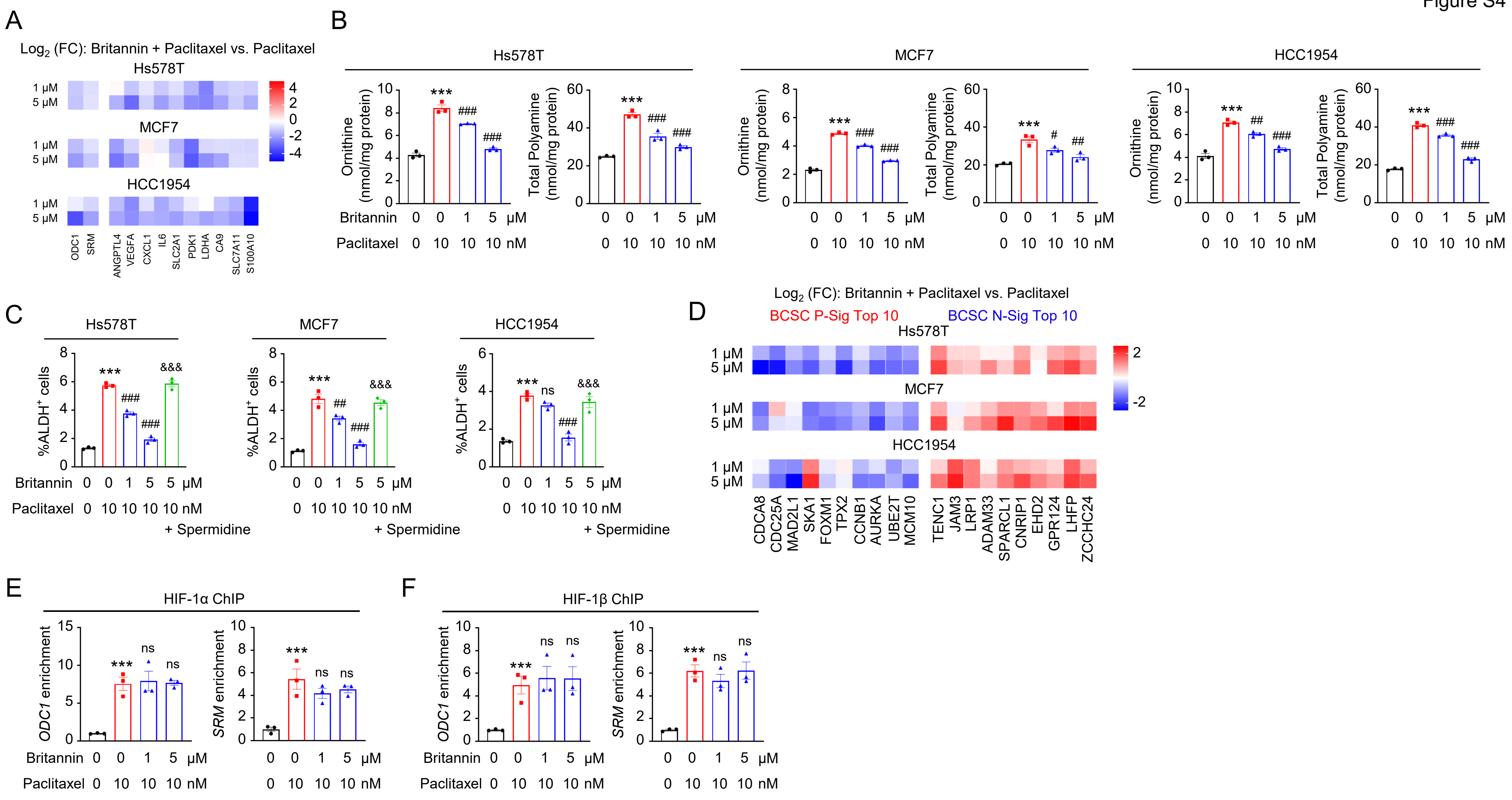
A
Log2 (FC): Britannin + Paclitaxel vs. Paclitaxel
Hs578T
MCF7
HCC1954
1 μM
5 μM
ODC1 SRM ANGPTL4 VEGFA CXCL1 IL6 SLC2A1 PDK1 LDHA CA9 SLC7A11 S100A10
B
Hs578T
MCF7
HCC1954
Ornithine (nmol/mg protein)
Total Polyamine (nmol/mg protein)
Britannin 0 0 1 5 μM
Paclitaxel 0 10 10 10 nM
C
%ALDH+ cells
Britannin 0 0 1 5 5 μM
Paclitaxel 0 10 10 10 10 nM
+ Spermidine
D
Log2 (FC): Britannin + Paclitaxel vs. Paclitaxel
BCSC P-Sig Top 10
BCSC N-Sig Top 10
CDCA8 CDC25A MAD2L1 SKA1 FOXM1 TPX2 CCNB1 AURKA UBE2T MCM10
TENC1 JAM3 LRP1 ADAM33 SPARCL1 CNRIP1 EHD2 GPR124 LHFP ZCCHC24
E
HIF-1α ChIP
ODC1 enrichment
SRM enrichment
F
HIF-1β ChIP
ns
